# In Vitro Fertilization Outcomes in Donor Oocyte Cycles Compared to the Autologous Cycles in the Poseidon 4 Group of Poor Ovarian Responders

**DOI:** 10.3390/medicina61020303

**Published:** 2025-02-10

**Authors:** Jelena Havrljenko, Vesna Kopitovic, Aleksandra Trninic Pjevic, Stevan Milatovic, Sandro Kalember, Filip Katanic, Tatjana Pavlica, Nebojsa Andric, Kristina Pogrmic-Majkic

**Affiliations:** 1 Ferona Fertility Clinic, Sarplaninska 19, 21000 Novi Sad, Serbia or jelenav@dbe.uns.ac.rs (J.H.); vesna.kopitovic@yahoo.com (V.K.); alex.trninic.pjevic@gmail.com (A.T.P.); sandrokalember@yahoo.com (S.K.); katanic.f@gmail.com (F.K.); 2 Department of Biology and Ecology, Faculty of Sciences, University of Novi Sad, Trg Dositeja Obradovica 2, 21000 Novi Sad, Serbia; tatjana.pavlica@dbe.uns.ac.rs (T.P.); nebojsa.andric@dbe.uns.ac.rs (N.A.); 3 Faculty of Medicine, University of Novi Sad, Hajduk Veljkova 3, 21000 Novi Sad, Serbia; milatstevan@gmail.com

**Keywords:** POSEIDON criteria, poor response, advanced age, gonadotropin dose, ovarian stimulation, oocyte donation

## Abstract

*Background and Objectives*: POSEIDON 4 (P4) patients face the most adverse outcomes among poor responders. Oocyte donation has overcome unsatisfactory live birth rates (LBRs) in P4 patients and has become an indispensable approach. However, many patients refuse oocyte donation despite poor live birth likelihood using autologous oocytes. This study aimed to determine clinical outcomes and live birth chances in P4 patients using autologous and donated oocytes. We also identified influencing factors of fertility outcome in P4 patients who underwent donor cycles. *Materials and Methods*: Retrospective data of 345 P4 patients who explored the first ovarian stimulation cycle (control group) were compared to 105 patients who failed to conceive and underwent repeated autologous ovarian stimulations with an increased starting gonadotropin dose and 100 unpregnant patients who received donated oocytes. Univariate analysis was used to identify prognostic factors of oocyte donation outcomes in P4 patients. *Results*: LBRs were significantly higher in the donor oocyte group. A higher number of retrieved and good-quality oocytes without differences in the blastocyst number and LBRs were found in the autologous patient group with adjusted gonadotropin dose compared to the control group. Univariate analysis showed that oocyte and embryo quality as well as blastocyst development had a positive impact on live birth in the donor patient group. *Conclusions*: Autologous in vitro fertilization (IVF) retreating and oocyte donation should be strongly advised for poor-prognosis P4 patients. Still, efforts in tailoring ovarian stimulation to obtain higher oocyte and embryo numbers in autologous cycles should be continued.

## 1. Introduction

In assisted reproduction, a large patient cohort consists of advanced-age women with diminished ovarian reserve [1]. A novel Patient-Oriented Strategies Encompassing IndividualizeD Oocyte Number (POSEIDON) classification of poor ovarian responders places those patients into the Poseidon 4 group, which is recognized as the group with the most adverse IVF outcomes [2]. P4 patients often face cycle cancellations or lower oocyte/embryo yield and, consequently, the lowest live birth rates among all Poseidon groups of poor responders [3]. According to the estimates, the proportion of the poor responder patients who appertain to the Poseidon 4 group could be up to 76% [1]. Despite the continuous efforts to enhance IVF outcomes, with the utilization of autologous oocytes, live birth rates among P4 poor responders are still unconvincing [2]. A more tailored approach, including conventional ovarian stimulation, dual stimulation, or natural cycles as well as common or adjusted gonadotropin doses, was proposed for treating this patient group [4]. Although some treatment options were shown to increase oocyte numbers significantly, the maturity and quality of obtained oocytes, as well as the implantation potential of developed embryos, could not be improved [5]. It has been evidenced that the commonest perpetrators of IVF outcomes, for advanced maternal age, are aneuploidies in oocytes [6]. Oocyte donation was introduced to overcome adverse IVF outcomes using autologous oocytes [7]. This approach allows a higher number of available oocytes, aneuploidy reduction, and, thus, higher chances for a live birth. Although previous disclosures emphasize a linear relationship between advanced maternal age and the risk of obstetrical complications [8], excellent live birth rates achieved with donated oocytes have resulted in a rise in oocyte donation cycles. Nowadays, pregnancies achieved when oocyte donation is applied represent about 5–7% of all embryo transfers [9]. However, the data that showed the advantages of oocyte donation for P4 patients who entered the oocyte donation cycles, or predictive factors for live births, are lacking. The most previous results were obtained from a very heterogeneous patient cohort.

Despite the outstanding live birth outcomes after oocyte donation, a majority of patients prefer IVF using their own oocytes over donation, as long as live birth chances are reasonable. Thus, increased maternal and neonatal complications, as well as a renouncing of biological maternity, lead to contemplating oocyte donation as a treatment of second choice [10]. For advanced maternal age (AMA) patients with reduced ovarian reserve who reject oocyte donation, the most widely applied strategy for increasing oocyte number is gonadotropin dose adjustment [11]. Although numerous reports have not shown an advantage in increasing ovarian response with higher doses [12,13], some researchers have found it beneficial [14,15]. In most of this research, higher gonadotropin doses might have resulted in a significant increase in the oocyte number, but this outcome did not improve the live birth rates. Some findings have even pointed to adverse effects of high gonadotropin doses. The study of Baart et al. showed that a minimal gonadotropin dose leads to the recruitment of more competent oocytes with fewer genetic errors and higher chances for live birth, compared to the increased gonadotropin doses, which may disturb oocyte–granulosa cell signaling and therefore negatively affect oocyte potential [16]. Contrarily, more recent studies found no correlation between the gonadotropin dose and aneuploidy rates [17].

In order to investigate IVF outcomes and the chances for live birth using autologous and donor oocytes in P4 patients, we conducted a study with two different approaches. Autologous oocyte utilization after gonadotropin dose adjustment and donated oocyte utilization were analyzed and compared to the IVF outcomes from the first autologous stimulation cycle. We also evaluated the reproductive outcomes of P4 patients who underwent oocyte donation to identify potential prognostic factors for oocyte donation in the P4 group.

## 2. Materials and Methods

### 2.1. Ethical Approval

The data used in this retrospective study were collected from the Ferona Fertility Clinic between 2020 and 2023, with the approval of Ferona Fertility Clinic’s Ethics Board (No 1220-1/2-23, approval date: 20 December 2023) and the consent of the University of Novi Sad, Faculty of Sciences Ethics Commission (No 0601-91/24-44, approval date: 13 September 2024), in accordance with the Declaration of Helsinki.

### 2.2. Patient’s Characteristics

The patients who took part in this study were ≥35 years and had <1 21 ng/mL Anti-Müllerian hormone (AMH) and <5 antral follicle count (AFC), thus belonging to the Poseidon 4 poor responder patient group according to a previously described classification [2]. No formal sample size calculation was performed. Serum AMH, FSH, and E2 levels were estimated on the second or third day of the menstrual cycle where AMH was measured using the ELISA assay with the minimum detectable level of 0.01 ng/mL, while FSH and E2 were measured using the CLIA assay. AFC was determined by transvaginal ultrasonography during the early follicular phase. Only patients who had available mature oocytes for fertilization and embryos for embryo transfer were included in the study. Patients with a severe male infertility factor (surgically retrieved spermatozoa, immotile spermatozoa, and <4% normal sperm morphology), untreated deleterious gynecological conditions, freeze-all, and preimplantation genetic testing (PGT) cycles, were excluded from the investigation. All eligible patients experienced at least one controlled ovarian stimulation (COS) cycle with a common starting gonadotropin dose and one COS cycle with an adjusted gonadotropin dose before committing to oocyte donation or continued autologous COS cycles with an increased gonadotropin dose.

### 2.3. Autologous Cycles

Patients dedicated to IVF cycles with their own oocytes received either the long gonadotropin-releasing hormone agonist or the flexible antagonist stimulation protocol [18]. Applied gonadotropins included human menopausal gonadotropin (hMG), recombinant FSH (rec FSH), or hMG+ rec FSH, with or without the addition of the clomiphene citrate during the early follicular phase. The decision on gonadotropin type was made according to the patient’s age and basal hormone level (FSH, E2, AMH) and the number of recruited follicles during the early phase of COS. The gonadotropin starting dose ranged from 75 to 150IJ during the first stimulation cycle and was increased up to 300IJ during the repeated stimulation cycles. The IVF procedure and embryo grading have been previously explained [19]. Briefly, 36 h after the ovulation trigger, oocytes were collected and fertilized using the intracytoplasmic sperm injection (ICSI) method. The embryo transfer (ET) and vitrification of surplus embryos were performed on Day 3 or Day 5. Embryo quality was determined according to blastomere characteristics (number, size, shape) and cytoplasmic features (fragmentation, multinucleation, vacuoles) for cleaved embryos or inner cell mass/trophoectoderm morphology (number and consistency of the cells) and expansion for the blastocyst stage. Oocyte quality was estimated in accordance with the European Society for Human Reproduction and Embryology (ESHRE) Atlas [20]. After ET or frozen ET (FET), patients received luteal support, and pregnancy outcomes were assessed according to the serum ß-hCG level and fetal heartbeat.

### 2.4. Donation Cycles

After confirming optimal endometrium thickness and progesterone level, imported vitrified donor oocytes were warmed using the Vitrolife Omni protocol [21]. One to two hours after warming, procedures including fertilization, embryo cultivation, oocyte and embryo quality evaluation, embryo transfer and vitrification of surplus embryos, luteal support, and pregnancy outcome confirmation were performed in the same manner as they were for the autologous cycles.

### 2.5. Main Outcome Analysis

The primary measures involved the number of retrieved, mature, good-quality, and fertilized oocytes, the number of developed and good-quality embryos, the number of blastocysts during the first stimulation cycles, and repeated cycles after gonadotropin dose adjustment using autologous oocytes. During the donation cycles, the primary measurements included the number of warmed, survived, mature, good-quality, and fertilized donated oocytes, the number of developed and good-quality embryos, and the number of blastocysts. The secondary measurements, during both the autologous and donation cycles, comprised clinical pregnancy, live birth, and miscarriage rates as well as cumulative clinical pregnancy, live birth, and miscarriage rates. Cumulative measurements involved fresh COS cycles and FET of previously cryopreserved surplus embryos after fresh ET.

### 2.6. Statistical Analysis

The statistical analysis was performed using SSPS software version 25.0. Differences in the primary and secondary outcomes were analyzed using the Student’s *t*-test and Pearson’s Chi-square test, where a *p*-value of 0.05 or lower was considered statistically significant. The effect of autologous and donor oocyte utilization on live birth rate (LBR) was evaluated using univariate logistic regression analysis.

## 3. Results

### 3.1. Study Population

**Figure 1:**
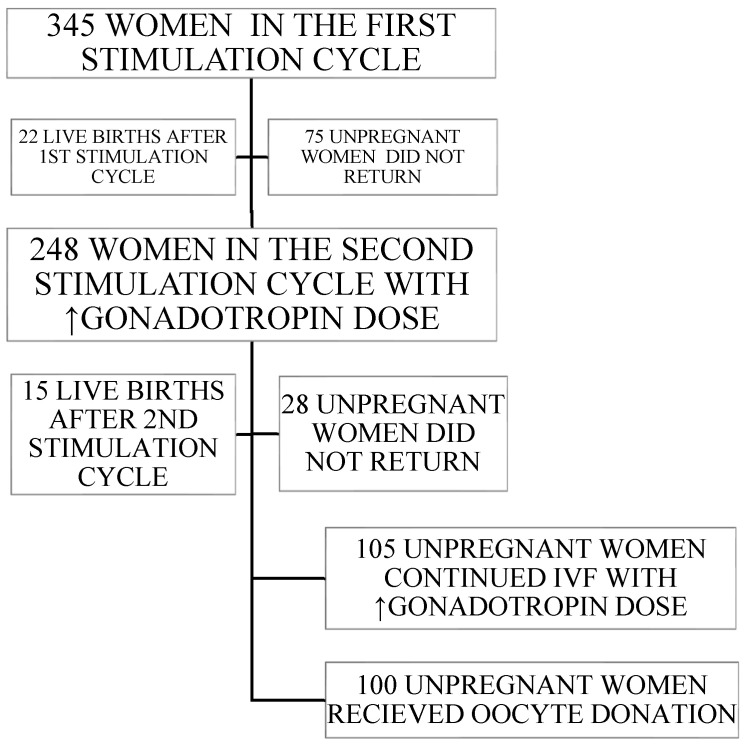
Flowchart of the patient inclusion.

Three hundred forty-five patients stratified into the P4 group of poor ovarian response according to their AMH, AFC, and age, who underwent their first controlled ovarian stimulation (COS) cycle, were assigned as the control group. Women who failed to conceive during the first COS cycle underwent the following COS cycle with an increased starting gonadotropin dose. Therefore, 105 unpregnant women rejected an oocyte donation and continued autologous IVF attempts with an increased gonadotropin dose (rIVF-iGD group) while 100 unpregnant women received donated oocytes (OD group). A flowchart of the patient distribution is presented in Figure 1, and the baseline characteristics of all analyzed patients are presented in Table 1.

The oldest patients with the highest serum FSH level were the ones who received donated oocytes, while patients in the control group were older than patients in the rIVF-iGD group (*p* < 0.001). There were no differences in the serum FSH level between the control and rIVF-iGD groups. The OD group had the lowest AMH level, but the differences were not significant between the three groups. The estradiol level was the lowest in the rIVF-iGD group, and the difference was significant compared to the control group (*p* < 0.02). The endometrium thickness was the most appropriate in the OD group compared to the other two groups (*p* < 0.001), while the thinnest endometrium was detected in the rIVF-iGD group (*p* < 0.001).

### 3.2. Autologous and Donor Oocyte Outcomes

The primary outcomes are detailed as follows (Table 2): Compared to the control group, rIVF-iGD had a significantly higher number of retrieved oocytes (*p* < 0.05) and number of good-quality oocytes (*p* < 0.05). The number of mature oocytes was higher in the OD group compared to the control group (*p* < 0.001), as was the number of good-quality and fertilized oocytes (*p* < 0.001). Unexpectedly, the number of mature oocytes was higher in the control group versus the rIVF-iGD group (*p* < 0.05). The number of developed embryos and blastocysts and the number of high-quality embryos were higher in the OD group compared to the control group (*p* < 0.001), while differences between the control group and the rIVF-iGD group were not found. Primary and secondary outcomes are shown in Table 2.

The secondary outcomes are detailed as follows: in terms of pregnancy outcomes, there were no differences between the control and the rIVF-iGD group in live birth and miscarriage rates. On the contrary, the OD group had significantly higher clinical pregnancy and live birth rates compared to the control group (*p* < 0.001), and there were no differences in the miscarriage rates (Table 2).

A comparison was also performed between the rIVF-iGD and the OD groups. The results revealed that the OD group showed a statistically significant increase in all primary and secondary outcomes (*p* < 0.001) in comparison to the rIVF-iGD group, except for the miscarriage rate where the differences were not noticed (Table 2).

To predict LBR using autologous and donated oocytes, a univariate logistic regression analysis was applied. It showed a significantly higher live birth likelihood with the utilization of donor oocytes, while there was no increase in live birth likelihood after repeated autologous IVF cycles with an increased gonadotropin dose (Table 3).

The univariate analysis was applied to compare the P4 patients who underwent oocyte donor cycles and had a live birth to those who did not. The results showed no correlation between the impact of age on live birth, the basal FSH level, and endometrial thickness. In contrast, the E2 level was significantly higher (*p* < 0.03) among women who had not achieved pregnancy. Regarding oocyte and embryo quality, in cycles that resulted in a live birth, oocyte and embryo quality were significantly higher (*p* < 0.04, *p* < 0.001) compared to the unsuccessful cycles. The development of a blastocyst also had a positive impact on live births and was significantly higher (*p* < 0.04) among women who accomplished pregnancy and had a live birth (Table 4).

## 4. Discussion

Management of advanced-age women with a reduced ovarian reserve and poor ovarian response is still a difficult challenge since this group is faced with increased age-related oocyte aneuploidy, higher cycle cancellation rates, and a higher need for multiple IVF attempts [22]. The treatment goal for these patients is to achieve at least one euploid blastocyst [23] by application of tailored individualized ovarian stimulation or, finally, oocyte donation. A recent investigation has estimated that the LBR for very-advanced-age poor responders using autologous oocytes is less than 5%, even after the transfer of a euploid embryo. Furthermore, the authors highlighted that no euploid embryos were developed beyond 46 years [24]. Evidence implicated that augmenting the dose of the gonadotropins in predicted poor responders, such as the Poseidon 4 group, does not enhance ovarian response, and they should move towards oocyte donation, as the most successful treatment solution [25].

This study aimed to evaluate the IVF outcomes of autologous and donor oocyte utilization in advanced-age poor responders with diminished ovarian reserve, confirming previous evidence where higher live birth rates were achieved through oocyte donation, compared to the live births in an autologous cycle [26].

In the group of patients who rejected oocyte donation and continued autologous IVFs, a gonadotropin dose adjustment has resulted in increased oocyte yield in comparison to the control group but has not influenced live births. In support of this finding, it was shown that higher gonadotropin doses may have an important influence on pregnancy achievement via endometrial thickness and receptivity. Previous reports revealed that higher gonadotropin doses may be associated with reduced endometrial receptivity due to the impaired hormonal milieu, where elevated progesterone levels caused by a higher FSH dose increase steroidogenic activity, but the direct effect of a higher FSH dose on the endometrium is unclear [14]. Kovac et al. identified gonadotropin dose adjustment as an independent negative predictor of endometrial thickness [27]. In line with this outcome, we found the thinnest endometrium in the rIVF-iGD patient group. Compared to both groups of autologous cycles, the LBR after oocyte donation surpassed live birth likelihood even after the gonadotropin dose was increased. Univariate logistic analysis asserted an increased live birth likelihood using donated oocytes, while repeated autologous cycles with an increased gonadotropin dose were unsupportable concerning improvements in live births. We found that women using donor oocytes were four times more likely to conceive than women using their own oocytes. This finding is confirmed by evidence that women’s age and oocyte aneuploidy are detrimental factors for IVF success [28]. Consequently, these prominent oocyte donation outcomes resulted in an exponential application of the procedure, especially in recent years since many clinicians consider autologous IVF among women >42 years with reduced ovarian reserve futile [29,30].

Although higher LBRs have not been accomplished with the gonadotropin dose adjustment, the results obtained in this study, considering an increased number of retrieved and good-quality oocytes, could present a possible treatment approach for patients refusing oocyte donation. After female age, the numbers of mature oocytes and developed embryos represent the crucial predictors of live birth [31]. A higher oocyte number leads to a higher embryo number, and, accordingly, it has been shown that at least three and four embryos were required for pregnancy achievement at the ages of 43 and 45, while live birth chance with only one embryo for very-advanced-age women is 2.5% [30]. Contrary to other reports, we found no improvements in the number of mature oocytes and developed blastocysts with an increased gonadotropin dose. A possible explanation for these unfavorable outcomes could be found in ovulation triggering. In this study, ovulation was induced with the administration of an hCG trigger, while recent research proposes a new “dual” approach where a combination of a GnRH agonist and an hCG, applied together for ovulation triggering, is associated with a higher number of mature oocytes after retrieval [32]. Several studies confirmed the association between an increased gonadotropin dose and a higher number of obtained oocytes in poor ovarian responders, while receiving a common daily dose of 150 international units (IU) resulted in an inadequate response [25]. Van Tilborg et al. found an increased number of retrieved oocytes when 225 IU and 450 IU of gonadotropins were applied, versus 150 IU (7.6 vs. 6.4, *p* < 0.002) [12]. A multicenter trial conducted to investigate mild ovarian stimulation strategy outcomes compared to the conventional strategy demonstrated a higher number of retrieved and mature oocytes, as well as developed embryos when patients received up to 450 IU of initial gonadotropin dose, versus 150 IU (5.0 vs. 3.3, 4.0 vs. 2.7, and 2.7 vs. 2.0, 95% CI) [33]. A more recent open-label randomized controlled trial also presented a higher number of obtained oocytes and available embryos when patients received 225–300 IU of gonadotropins, instead of a common 150 IU dose [14]. Still, in line with our results, this research found prosperity of an increased gonadotropin dose only in oocyte/embryo number, but it did not find improvements in the LBRs. A study by Baker et al. involving >650 000 IVF cycles discovered a negative correlation between gonadotropin dose and LBRs [34]. According to all of this evidence, we could assume that an increased gonadotropin strategy for achieving a higher ovarian response could be applicable only in particular occasions, such as preimplantation genetic testing (PGT). Some reports suggest PGT for the selection of the most potential euploid embryo to increase the live birth likelihood in poor advanced-age responders; therefore, generating a large embryo cohort should be required [35,36]. Considering all of the aforementioned, evidence for increasing the gonadotropin dose over 150 IJ to enhance pregnancy outcomes is inconclusive, and precaution should be strongly recommended in the implementation of this strategy in Poseidon 4 patients who dismiss oocyte donation.

In this research, cumulative live birth rates using previously vitrified donor oocytes reached 24%, comparable to other investigations [37,38,39,40]. However, studies report up to 50% or higher live births using both vitrified and fresh donor oocytes. The ESHRE monitoring of the available data in 2018 reported 49.6%/44.9% pregnancy rates per ET using fresh/frozen donor oocytes, among 36 938 donation cycles [41]. A prospective case–control study in 2018 compared vitrified donor oocyte and autologous oocyte outcomes where the LBR per embryo transfer in the donor group was 46% [42]. A large retrospective study of 40 485 donor cycles showed a 53.3% live birth rate using commercial oocyte banks and 55.4% LBR with recruited oocyte donors after single ET, while double ET had a further increased LBR [43]. Some research has indicated increasing differences in live births given that cryopreservation and the thawing of oocytes could negatively affect implantation potential due to the formation of ice crystals that damage oocyte integrity [44]. Moreover, events occurring during the vitrification process, such as an alteration in gene expression, a reduction in mitochondrial DNA content, impaired calcium ionophore pathways, and increased reactive oxygen species, might also affect oocyte survival after warming, as well as the developmental competence of survived oocytes [45]. However, contrary to studies showing lower LBRs using vitrified donor oocytes compared to fresh ones [46], in 2021, the American Society for Reproductive Medicine (ASRM) reported no significant differences in clinical pregnancy rates between fresh and vitrified donor oocytes. Still, it emphasized the need for further studies concerning the evaluation of live births [47]. Thus, in vitrified donor programs, the most important prerequisite for success is to establish efficient freezing and warming techniques [37].

Another crucial factor for maximizing LBR in donor cycles with vitrified oocytes is a sufficient oocyte number assigned to the recipient. In agreement with an analysis of >6000 donor cycles with vitrified oocytes where CLBR reached 15.8% with five oocytes and 32% with eight oocytes [48], in this study, a batch containing six or seven vitrified oocytes was submitted to each patient. Furthermore, the developmental stage at embryo transfer has also been proposed as an influence on clinical outcomes [9]. Significant improvements in pregnancy rates were achieved when blastocyst-stage embryo transfers were employed [49,50]. Contrary to these disclosures, for the most part, we performed embryo transfers on Day 3 since the overall number of developed and good-quality embryos was insufficient. We assume that this outcome could be a consequence of low oocyte quality observed after warming. In this respect, Karagianni et al. reported slower first cell division of embryos derived from vitrified donor oocytes compared to the fresh donor oocytes; however, the authors indicated a minimal impact of the detected changes on subsequent embryo development [51].

Another interesting result of this study comes from the comparison of the P4 patients who underwent an oocyte donor cycle and had a live birth and those who did not. These two subgroups of P4 patients did not show an age difference and used vitrified donor oocytes, thus eliminating the age difference and the impact of fresh vs. vitrified oocytes on the success of a donor cycle. The univariate analysis data for these two groups suggest that oocyte quality, good embryo quality, and blastocyst development play a critical role in live birth among P4 patients. This result could, potentially, be explained by data showing that even in centers with benchmark survival rates, certain vitrification/warming procedures inevitably fall below competence levels, even reaching <50% survival, because of the biological variability in oocyte quality, technical variability, and the manual aspect of the cryopreservation process, among other factors [52]. Moreover, a recent paper has pointed out the efficacy of imported vitrified oocytes concerning the cryobank of origin. A comparison has shown significant differences in survival rates (70.1%, 89.0%, and 81.0%) and CLBR (31.2%, 56.0%, and 50.8%) between three different cryobanks, suggesting reduced oocyte viability from one particular cryobank [53]. Therefore, improvements in the vitrification/warming protocol are required to increase outcomes in poor responders who underwent a donor oocyte cycle. In addition to this, our investigation also confirmed previous associations between elevated basal estradiol levels on Day 2 and lower live birth rates as a consequence of low endometrial receptivity caused by high estradiol levels [54,55,56].

To our knowledge, this study may be joined to only a few previous comparisons of autologous and donated oocyte utilization among Poseidon 4 patients. It also provides critical information that could help clinicians identify patients with very low prognoses and individualize their treatment according to their specific needs. This research has important clinical implications since it reports outcomes after one stimulation cycle and a repeated cycle and therefore could establish realistic expectations. Furthermore, results that refer to autologous cycles with increased gonadotropin dose significantly contribute to general opinions regarding an unjustified utilization of this approach for better IVF outcomes. In addition, this research reveals an association between patient or cycle characteristics and low IVF outcomes. Another strength of the study lies in the exclusion of the male factor which could affect the outcome. The results obtained in this study might help patients to prepare themselves emotionally and financially for autologous treatment as well as balance the desire for a genetic link with the low success rates of repeated autologous cycles and moving towards oocyte donation. Although our investigation provides useful information for treating very poor ovarian responders, it is limited due to its retrospective nature, lack of randomization, and the low number of patients included in the study after dropout.

## 5. Conclusions

In relation to a very low live birth likelihood in Poseidon 4 patients, oocyte donation should be encouraged, especially in cases of very poor prognosis. In addition, to enhance live birth rates using donated oocytes, upgrades in vitrification and warming protocols are still required. On the other hand, research leading to improvements in autologous treatment approaches should not be abandoned given that the majority of patients do not want to renounce a genetic relation to their offspring.

## Figures and Tables

**Table 1:** Baseline characteristics of the patients.

	Autologous Oocytes		Donor Oocytes	*p*-Value
	1st IVF (Control Group)	rIVF-iGD Group	OD Group	
Total number	343	105	100	-
Female age (years)	40.86 ± 2.99	40.32 ± 2.46	42.91 ± 3.97	<0.001
Donor age (years)	-	-	28.01 ± 3.17	-
AMH level (ng/mL)	0.49 ± 0.29	0.55 ± 0.69	0.46 ± 1.19	NS
FSH level (mIU/mL)	11.79 ± 6.97	12.19 ± 5.80	21.59 ± 18.49	<0.001
E2 level (pg/mL)	99.57 ± 110.45	80.64 ± 72.63	90.02 ± 119.31	<0.02
Endometrial thickness (mm)	9.27 ± 1.90	9.15 ± 1.87	10.2 ± 2.58	<0.001
Oocyte survival rate	-	-	91.04%	-

Results are presented as mean ± SD or percentage; *p*-value ≤ 0.05 is considered statistically significant; confidence intervals: 95% (*p* ≤ 0.05), 99% (*p* ≤ 0.01). Abbreviations: rIVF-iGD group (continued autologous IVF attempts with an increased gonadotropin dose); OD group (oocyte donation cycles); AMH (Anti-Müllerian hormone); FSH (follicle-stimulating hormone); E2 (estradiol); NS—not statistically significant; SD—standard deviation.

**Table 2:** Primary and secondary IVF outcomes in autologous and donation cycles.

	Autologous Oocytes		Donor Oocytes
	Control Group	rIVF-iGD Group	OD Group
Total patient number	343	105	100
Total number of retrieved oocytes	1070	333	603
Number of retrieved oocytes or imported oocytes	3.12 ± 2.09	3.17 ± 2.23 *	6.09 ± 0.41
Number of mature (MII) oocytes	2.35 ± 1.54	2.14 ± 1.72 *	5.55 ± 0.87 **^#^
Number of good-quality oocytes	1.74 ± 1.61	1.79 ± 1.67 *	3.30 ± 2.52 **^#^
Number of fertilized oocytes	2.06 ± 1.31	2.05 ± 1.34	4.54 ± 1.22 **^#^
Number of developed embryos	1.98 ± 1.28	1.97 ± 1.26	3.66 ± 1.40 **^#^
Number of high-quality embryos	1.10 ± 1.06	1.11 ± 1.07	1.69 ± 1.48 **^#^
Number of developed blastocysts	0.07 ± 0.44	0.06 ± 0.41	0.24 ± 0.81 **^#^
Number of transferred embryos	1.68 ± 0.78	1.67 ± 0.64	1.69 ± 0.63
Clinical pregnancy rate	44 (12.83%)	13 (12.40%)	24 (24.00%) **^#^
Live birth rate	30 (8.75%)	9 (8.60%)	20 (20.00%) **^#^
Miscarriage rate	14 (4.08%)	4 (3.80%)	4 (4.00%)
Cumulative clinical pregnancy rate	47 (13.70%)	13 (12.40%)	28 (28.00%) **^#^
Cumulative live birth rate	25 (9.62%)	9 (8.60%)	24 (24.00%) **^#^
Cumulative miscarriage rate	14 (4.08%)	4 (3.80%)	4 (4.00%)

Results are presented as mean ± SD and percentage. * *p* < 0.05 vs. control group; ** *p* < 0.001 vs. control group; ^#^ *p* < 0.001 vs. rIVF-iGD group; *p*-value ≤ 0.05 is considered statistically significant; confidence intervals: 95% (*p* ≤ 0.05), 99% (*p* ≤ 0.01). Abbreviations: rIVF-iGD group (continued autologous IVF attempts with an increased gonadotropin dose); OD group (oocyte donation cycles); SD—standard deviation.

**Table 3:** Univariate logistic analysis for live birth prediction.

	Odd Ratio (OR)	Confidence Interval (95%)	*p*-Value
1st autologous IVF cycle (control group)	**Reference**		
Repeated autologous IVF (rIVF-iGD group)	0.01	0.00–0.01	0.99
Donation cycles (OD group)	2.63	1.01–7.62	0.05

*p*-value ≤ 0.05 is considered statistically significant.

**Table 4:** Univariate analysis of the live birth prognostic factors in oocyte donation cycles.

Independent Variables (Mean ± SD)	Live Birth (*n* = 24)	No Live Birth (*n* = 76)	*p*-Value
Age (years)	42.08 ± 4.55	43.13 ± 3.82	0.27
Basal FSH (mIU/mL)	25.46 ± 17.81	19.77 ± 18.16	0.19
Basal E2 (pg/mL)	56.60 ± 61.40	101.92 ± 133.46	<0.03
Endometrial thickness (mm)	10.15 ± 1.16	10.21 ± 2.94	0.93
Number of good-quality oocytes	4.13 ± 2.15	2.90 ± 2.58	<0.04
Number of good-quality embryos	2.75 ± 1.51	1.38 ± 1.32	<0.001
Number of developed blastocysts	0.54 ± 1.18	0.16 ± 0.65	<0.04

*p*-value ≤ 0.05 is considered statistically significant; confidence intervals: 95% (*p* ≤ 0.05), 99% (*p* ≤ 0.01).

## Data Availability

All data involved in this work will be made available by the corresponding author upon request (kristina.pogrmic@dbe.uns.ac.rs).
